# Causes, risk factors, and complications of accidental intra-arterial administration of medications in a children’s hospital: a case series

**DOI:** 10.1186/s40981-024-00728-x

**Published:** 2024-09-02

**Authors:** Yuki Kunioku, Rie Minoshima, Yutaro Chida, Shinichi Nishibe

**Affiliations:** Tokyo Metropolitan Children’s Medical Center, 2-8-29 Musashidai, Fuchu-Shi, Tokyo, 184-8561 Japan

**Keywords:** Accidental arterial cannulation, Anesthetic, Pediatric, Intra-arterial drug injection, Peripheral venous cannulation

## Abstract

**Background:**

Accidental intra-arterial administration of a medication can lead to serious iatrogenic harm. Most studies have discussed single cases of accidental intra-arterial administration of a medication, but only a few have described multiple cases occurring in a single, pediatric hospital setting.

**Methods:**

The subjects were pediatric patients with an accidental intra-arterial administration of a medication. After obtaining approval from the institutional review board, the relevant cases were extracted from incident reports submitted to the patient safety office of the study center between November 2016 and April 2023.

**Results:**

A review of 18,204 incident reports yielded 10 cases (patient age: 27 days to 13 years) of accidental intra-arterial administration of a medication. The most common site of the cannulation was the dorsum of the foot followed by the dorsum of the hand. The medications administered were narcotics, sedatives, muscle relaxants, antibiotics, and crystalloids. No serious adverse events occurred after injection. In some cases, the accidental arterial cannulation was not discovered immediately (53 min to 26 days). Seven patients had difficult intravenous access; in two of these, ultrasound-guided peripheral venous cannulation was used.

**Conclusions:**

We experienced 10 cases of accidental intra-arterial administration of a medication. The dorsalis pedis artery and the radial artery around the anatomical tobacco socket were common sites of unintentional arterial cannulation. Difficult intravenous (IV) access may be associated with unintentional arterial cannulation. If IV access is difficult or the free IV drip is sluggish, strict vigilance and repeated confirmation are needed to prevent unintentional arterial cannulation.

## Background

Accidental intra-arterial administration of a medication can lead to serious morbidities, such as paresthesia, chronic pain, and tissue necrosis [1]. This error is caused by either of two mechanisms: inadvertent cannulation to an artery instead of a vein or an accidental administration of a medication into an established arterial cannula for invasive blood pressure monitoring [2]. The proximity of normal vascular structures, aberrant vasculature, difficult procedures, and iatrogenic errors all contribute to the unintentional cannulation of arteries in the process of establishing intravenous access [1].

To date, there are few reports describing multiple cases in a single, pediatric clinical setting from the standpoint of patient safety; most studies have discussed individual cases of accidental intra-arterial administration of a medication. The present study analyzed data from incident reports submitted to a patient safety office to identify the causes, risk factors, and complications associated with accidental intra-arterial administration of a medication in a single, pediatric hospital.

## Methods

The present retrospective cohort study was approved by the Institutional Review Board at Tokyo Metropolitan Children’s Medical Center (approval no.: 2022b-84; date: 2022 November 30). Relevant cases were extracted from incident reports submitted to the patient safety office of the institute between November 2016 and April 2023 and were comprehensively reviewed for information relevant to “procedure,” “arterial puncture,” “peripheral venipuncture,” and “drugs” under “intravenous infusion and blood transfusion” and “surgery, anesthesia, procedures, laboratory tests, and medical care.” The patients’ age, cannulation site, health center, medication(s) involved, circumstances leading to the discovery of the incident, time from cannulation to discovery, morbidities, outcome, and the background of the incident were extracted from electronic medical records.

## Results

In 18,204 incident reports, there were 10 cases of accidental intra-arterial administration of a medication (Table 1). The patients’ age ranged from 27 days to 13 years and 8 months, and the cannulation sites included the dorsum of the hand (*n* = 2), the dorsum of the foot (*n* = 6), a lower limb (*n* = 1), and axilla (*n* = 1). Four cases of unintentional drug administration occurred in the general ward, three occurred in the operating room (OR), and one occurred in a pediatric intensive care unit (PICU), neonatal intensive care unit (NICU), and the emergency room (ER) each. The medications administered were narcotics, sedatives, muscle relaxants, antibiotics, and crystalloids. The most common clues to the discovery of an accidental intra-arterial injection were the absence of fluid freely dripping into the intravenous (IV) drip chamber under gravity (free IV drip), presence of blood pulsation or reverse blood flow within an IV line (flashback), and discoloration of the skin surrounding the cannulation site. One case was discovered incidentally from a radiographic image. The shortest time from cannulation to the discovery of the error was 53 min, while the longest was 26 days, 2 h, and 22 min. All accidental arterial cannulations were immediately removed on discovery. None of the patients suffered any morbidities.

**Table 1:** Ten cases of accidental intra-arterial administration of a medication

Case	Cannulation site	Age	Place of occurrence and discovery	Medication involved	Clues to discovery	Time from cannulation to discovery	Sequelae	Presumed causes and backgrounds of misplacement	Presumed causes and backgrounds of administration
1	Dorsalis pedis artery (left)	3 months	PICU	Midazolam, morphine, 5% glucose	Pulsatile flashback within IV tube	7 h 50 min	No deficit	Difficult IV access, US-guided peripheral venous access, judgement error	Poor flashback at the start of administration, non-bright-red flash of blood at cannulation, use of infusion pump
2	Subclavian artery (left)	27 days	NICU	Heparin, 5% glucose, normal saline	Contrast-enhanced CT	26 days, 2 h 22 min	No deficit	Difficult IV access, PI catheter, trisomy 18, VLBW	Misdiagnosis from X-ray (diagnosed as PI in the subclavian vein)
3	Ulnar artery (branch) (left)	4 years 5 months	Ward, OR	Fentanyl, rocuronium, phenylephrine, propofol, lidocaine, 5% glucose-acetated Ringer's	Pulsatile flashback within IV tube confirmed by BGA	17 h 49 min	No deficit	Normal vascular anatomic proximity, lack of anatomical knowledge	Confirmation of free IV drip at the start of administration, non-pulsatile flashback at the start of administration, no resistance to drug administration in the use of infusion pump
4	Dorsalis pedis artery (left)	2 years	OR	Fentanyl, remifentanil, propofol, rocuronium, atropine sulfate, cefazolin, 5% glucose-acetated Ringer’s	Absence of free IV drip confirmed by BGA	2 h 23 m	No deficit	Difficult IV access, overconfidence	Confirmation of free IV drip at the start of administration, non-pulsatile flashback at start of administration, backflow prevention valve on IV line
5	Dorsalis pedis artery (left)	8 years 3 months	Ward	Maintenance intravenous fluid	Absence of free IV drip, pulsatile flashback within IV tube	53 min	No deficit	Difficult IV access, US-guided peripheral venous access, heparin lock immediately after cannulation	Confirmation of free IV drip before drug administration IV line locked with heparin before administration
6	Dorsalis pedis artery (left)	7 m	Ward	Vancomycin, maintenance IV solution	Pulsatile flashback within IV tube	60 min	No deficit	Difficult IV access, normal vascular anatomical proximity, overconfidence, premature closure	Non-pulsatile flashback at the start of administration, poor flashback at the start of administration
7	Radial artery (right)	5 years 7 months	ER, ward	Ampicillin, 5% glucose-acetated Ringer’s	Pulsatile flashback within IV tube, skin discoloration around cannulation site	16 h 23 min	No deficit	Normal vascular anatomic proximity, lack of anatomical knowledge, overconfidence, premature closure	Recognition of free IV drip at the start of administration
8	Dorsalis pedis artery (right)	3 months	Ward, OR	Maintenance intravenous fluid	Absence of free IV drip, pulsatile flashback within IV tube	1 h 25 min	No deficit	Difficult IV access, judgement error, overconfidence	Smooth injection without resistance, backflow prevention valve on IV line
9	Dorsalis pedis artery (right)	4 years 11 months	Ward	Gentamicin, maintenance intravenous fluid, normal saline	Absence of free IV drip, pulsatile flashback within IV tube	1 day, 14 h 15 min	No deficit	Difficult IV access, cannulation in a dark room	Zebra retreat, overconfidence (intra-arterial cannulation was recognized as a rare event)
10	Anterior tibial artery (left)	13 years 8 months	Previous center, ER	Midazolam, rocuronium, normal saline	Absence of free IV drip, pulsatile flashback within IV tube	4 h 22 min	No deficit	Difficult IV access	IV line established in previous medical center, lack of confirmation, failure to check

*Abbreviations*: *OR* Operating room, ER Emergency room, *US* Ultrasound, *PI* Peripherally inserted central catheter, *IV* Intravenous flashback, regurgitant blood flow from cannula, *BGA* Blood gas analysis

Seven patients had difficult intravenous access. Ultrasound (US)-guided peripheral venous cannulation was used in two patients. In addition, the presence of free IV drip was confirmed in four patients, no pulsatile flashback was observed in four patients, and a backflow prevention valve was used in two patients at the start of drug administration. An infusion pump was used in all the patients. There were no cases of accidental administration of a medication via an established intra-arterial route for invasive blood pressure monitoring.

Eight of 10 incident reports of unintentional arterial cannulation were filed by physicians. Among these, six physicians pointed out human error as a contributing factor.

## Discussion

The most common site (six cases) of accidental arterial cannulation was the dorsum of the foot. The dorsal cutaneous vein is often chosen for peripheral venous access in children because it is superficially located and can therefore be palpated and inspected readily. Cutaneous veins, in contrast to deep veins, are situated close to the skin and travel without an accompanying artery or nerve. However, the dorsalis pedis artery is superficially located on the medial side of the foot, especially in the first metatarsal space, and its branches travel along the dorsal venous arch of the foot [3, 4]. Therefore, the risk of accidental arterial cannulation at the dorsum of the foot is high. The next most common site (two cases) of accidental arterial cannulation was the dorsum of the hand. On the radial side of the dorsum of the hand, the radial artery and the first dorsal metacarpal artery travel along the anatomic tobacco fossa (tabaciére) and the first dorsal interosseous muscle. These arteries often travel beneath the proximal part of the dorsal venous arch of the hand. Moreover, the second and third dorsal metacarpal arteries can cross under the distal part of the dorsal venous arch of the hand [5, 6]. Accordingly, it should be noted that venous cannulation around these sites can accidentally involve the artery.

In the present study, anesthetics (midazolam, rocuronium, propofol, heparin, phenylephrine, lidocaine, atropine), narcotics (morphine, fentanyl, remifentanil), antibiotics (cefazolin, vancomycin, ampicillin, gentamicin), and crystalloids (5% glucose solution, normal saline, 5% glucose acetate Ringer’s solution, 1% glucose acetate Ringer’s solution, and maintenance intravenous fluids) were unintentionally administered via an artery. The delivery of certain medications via arterial access can have clinically important sequelae, including paresthesia, severe pain, motor dysfunction, compartment syndrome, gangrene, and limb loss [1]; however, none of the medications administered in this study caused serious complications. Thiopental and lipophilic diazepam, both of which are strongly alkaline and lipophilic agents, reportedly cause serious complications, such as tissue necrosis, when administered via an artery [7, 8], although the mechanism by which these substances cause symptoms of greater severity than either hydrophilic fentanyl or rocuronium is unknown [1]. Propofol is another lipophilic anesthetic related to unique injection pain, but no serious symptoms have been reported in its intra-arterial administration [9, 10]. The results of the present study suggested that the accidental intra-arterial administration of anesthetics used in pediatric medicine, other than certain drugs, such as thiopental and diazepam, usually does not induce serious tissue damage unless extravasation occurs.

Certain cases of accidental intra-arterial cannulation were detected only after a considerable lapse of time. Children and anesthetized patients are unable to complain about local pain or discomfort, which often appear as the initial symptoms of an accidental arterial injection of a medication. Moreover, the use of an infusion pump and backflow prevention valve might hinder the detection of an accidental intra-arterial infusion because both devices make free IV drip or pulsatile regurgitation of blood difficult to observe. As with an extravasation injury, which is another issue related to patient safety in children’s hospitals, the periodic confirmation of free IV drip is important to guard against accidental arterial cannulation. It is well-known that the occlusion alarm of the infusion pump cannot detect extravasation or accidental intra-arterial cannulation in children. The Japanese Industrial Standard (JIS) dictates that the occlusion alarm pressure of an infusion pump be set higher than 0.4 kgf/cm^2^ [11]. Given that the systolic blood pressure of children is about 80–125 mmHg (the value in kgf/cm^2^ is 0.10–0.16 kgf/cm^2^), the occlusion alarm is ineffective. Similarly, arterial pressure is lower in children than in adults, thus possibly attenuating pulsatile flashback and causing a sluggish free IV drip in cases of accidental intra-arterial cannulation and drug administration.

The utility of ultrasonography (US)-guided peripheral venous cannulation in children with difficult intravenous access has been demonstrated [12, 13]. On the other hand, US-guided peripheral venous access is reportedly a risk factor of extravasation because the technique is often indicated in patients requiring multiple attempts at cannulation due to difficult venous access. A potential mechanism of extravasation in a patient with difficult venous access is the dislodgement of a catheter of inadequate length in peripheral venous cannulation guided by US, which allows access to deeper or subfascial veins [14]. In the present study, US-guided peripheral venous cannulation was used in two cases of accidental intra-arterial cannulation to the dorsalis pedis artery. As mentioned above, in addition to the anatomical proximity of the artery to the vein at the dorsum of the foot, the effect of the tourniquet must be considered. In adults, the appropriate tourniquet pressure for peripheral venous cannulation is 40–95 mmHg [15]. When a tourniquet is applied to the extremities for vascular access, the vein, like the artery, can be resistant to compression with the US probe. It is important to note that arterial pressure in neonates and infants is low, and the tourniquet can easily diminish the arterial pulsation. Moreover, if the compression with a US probe is too strong, it can collapse the artery to the extent that it resembles a vein. Therefore, carefully distinguishing between venous and arterial structures before cannulation should be done not only by compressing the vein and confirming the lack of pulsatility but also, and more importantly, by confirming the venous flow using pulsed wave Doppler [16].

The years of experience of the physicians who unintentionally placed an intra-arterial cannula ranged from 5 to 22 years, suggesting that such incidents are rare but can occur even among experienced physicians. In this study, six of eight physicians pointed to human error, such as overconfidence, premature closure, or wrong judgement rather than inexperience or technical failure as the factors contributing to intra-arterial cannulation in their incident report [17, 18]. This may indicate that physicians, including anesthesiologists, have a poor awareness of accidental intra-arterial injection. Accidental intra-arterial injection is always a potential complication of infusion therapy; thus, raising awareness of the difficulty of recognizing an arterial misplacement during cannulation or drug administration in children can help prevent it. If IV access is difficult or a free IV drip is sluggish, strict vigilance and repeated confirmation are needed to prevent unintentional arterial cannulation. If the cannulation is doubtful, injection of saline through the cannula is recommended to induce blanching as a way to confirm intra-arterial cannulation [19].

In the present study, there were no cases of intra-arterial drug administration via an established intra-arterial route for invasive blood pressure monitoring. The inadvertent intra-arterial injection of drugs may be difficult to prevent especially in the OR, PICU, NICU, and ER because patients often receive multiple infusions through numerous ports. According to the institutional patient safety protocol, intra-arterial lines and ports for invasive arterial pressure monitoring must be colored red to distinguish them from venous lines. Also, the configuration of the ports of arterial lines are completely different from that of venous lines. These measures may have limited the occurrence of intra-arterial drug administration.

To conclude, we experienced 10 cases of accidental intra-arterial administration of medications. From the anatomical standpoint, the dorsalis pedis artery and the radial artery around the anatomical tobacco socket are particularly vulnerable to unintentional arterial cannulation. The use of an infusion pump or backflow prevention valve might delay the detection of an unintentional arterial cannulation. Difficult intravenous access, including US-guided peripheral venous access, may be associated with unintentional arterial cannulation. If IV access is difficult or a free IV drip is sluggish, strict vigilance and repeated confirmation are needed to prevent unintentional arterial cannulation.

## Data Availability

The datasets used and analyzed during the current study are available from the corresponding author upon reasonable request.

## Abbreviations

IV: Intravenous
US: Ultrasonography
OR: Operating room
PICU: Pediatric intensive care unit
NICU: Neonatal intensive care unit
ER: Emergency room
